# Bacterial Inactivation on Concrete Plates Loaded with Modified TiO_2_ Photocatalysts under Visible Light Irradiation

**DOI:** 10.3390/molecules24173026

**Published:** 2019-08-21

**Authors:** Magdalena Janus, Ewelina Kusiak-Nejman, Paulina Rokicka-Konieczna, Agata Markowska-Szczupak, Kamila Zając, Antoni W. Morawski

**Affiliations:** 1 Department of Sanitary Engineering, Faculty of Civil Engineering and Architecture, West Pomeranian University of Technology, Szczecin, al. Piastów 50, 70-311 Szczecin, Poland; 2 Institute of Inorganic Chemical Technology and Environment Engineering, Faculty of Chemical Technology and Engineering, West Pomeranian University of Technology, Szczecin, ul. Pułaskiego 10, 70-310 Szczecin, Poland

**Keywords:** photoactive concrete, photocatalysis, titanium dioxide, modified TiO_2_, *Escherichia coli* inactivation

## Abstract

The antibacterial activity of concrete plates loaded with various titania photocatalysts was investigated. The target in bacteria testing was *Escherichia coli* K12. The presence of photocatalysts in the concrete matrix at a dose of 10 wt.% improved the antibacterial properties, which became significant depending on the type of the added photocatalyst. Total inactivation of *E. coli* irradiated under artificial solar light was observed on the concrete plates loaded with the following photocatalysts: TiO_2_/N,C_MeOH_-300, TiO_2_/N,C_EtOH_-100, TiO_2_/N,C_isoPrOH_-100 and TiO_2_/N-300. The modified Hom disinfection kinetic model was found as a best-fit model for the obtained results. The presence of nitrogen and carbon in the photocatalysts structure, as well as crystallite size, surface area and porosity, contributed to the increase of antibacterial properties of concrete plates.

## 1. Introduction

The building materials industry is amongst the fastest-growing field of study. It is very well known that concrete products are the most widely used construction materials. In order to ensure the appropriate health of customers, suitable legal requirements, new quality standards, and methods of production of the modern eco-friendly building materials are continually being developed. However, the availability of products with antimicrobial properties is a relatively new phenomena (more than 2000 in 2014) [1,2]. Due to their potential applications as antimicrobial surfaces that inhibit the growth of bacteria, viruses and mould fungi, these materials can find applications in various settings, including medical clinics, industry, swimming baths and households. According to the World Health Organization (WHO), waterborne diseases cause more than 3.4 million deaths annually, mostly among young children in developing countries. It appears that to prevent water-associated infections, essential use of antibacterial materials is at water treatment plants. Development of a water storage system to provide a predisinfection process or disinfecting water storage can reduce health problems significantly. It will be of particular importance in the developing world and areas particularly vulnerable to water deficits where water is collected from outside sources, such as snow and rain. 

In this context, the additiion of titanium dioxide to construction materials shows high potential [3,4,5]. TiO_2_-coated or TiO_2_-loaded building products mostly indicate enhanced self-cleaning, anti-fogging, or anti-odour properties [6,7]. Unfortunately, the excitation of TiO_2_ with ultraviolet radiation (TiO_2_ absorption edge below 400 nm) is regarded as among the most severe disadvantage. On the other hand, the building structures are usually exposed to sunlight containing UV light. From a practical point of view, the study of the photoactivity of building materials containing titanium dioxide, which are activated by UV light, is also an interesting scientific aspect that was discussed by Petrella et al. [8,9]. The most effective solution is to modify the structure of TiO_2_ with non-metal or transition metal ions [10,11,12]. The presence of dopants in the TiO_2_ crystalline matrix significantly influences its photoactivity, thereby decreasing the deactivation of the photocatalysts and suppressing the charge carriers recombination [13]. It was also shown that doping of pristine titania with non-metal ions causes the increase of reactive oxygen species (ROS) formed on the surface of TiO_2_ [14,15]. All of them caused direct oxidation of organic pollutants and evoked oxidative stress, which can lead to bacterial cell death [16]. 

Because in the literature there is still a lack of experimental results focused on the direct interaction between concretes supplemented with modified titania and bacteria, we tried to explain the mechanism based on obtained results. According to Markov et al. [17], the main disadvantage of the mentioned method is that the reactive oxygen species are probably deactivated before traversing the layer due to their short life. The aim of this study was to investigate the antibacterial properties of concretes loaded with commercial titania AEROXIDE^®^ TiO_2_ P25, nitrogen (N) modified or nitrogen and carbon (C,N) co-modified titania photocatalysts. Carbon (from methanol, ethanol and isopropanol) and nitrogen (form gaseous ammonia) modifications of commercial TiO_2_ were carried out at 100, 300 and 600 °C. Gram-negative *Escherichia coli* strain K12 was used in all experiments. This bacterium commonly lives in the intestines of people and animals but can also be presented in the indoor environment [18]. 

## 2. Results and Discussion

**Figure 1:**
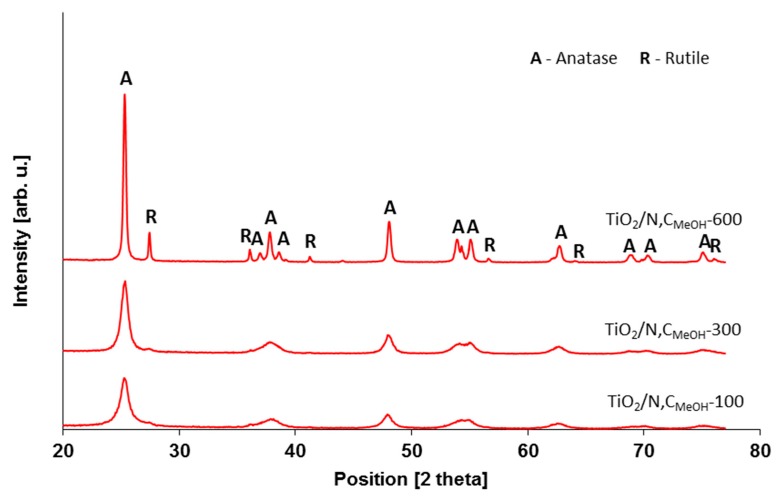
XRD patterns of photocatalysts modified with e.g., methanol at different temperatures.

**Figure 2:**
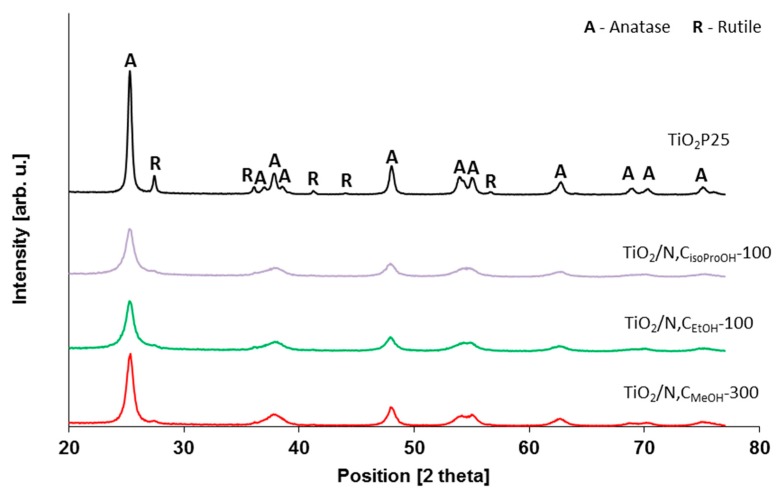
XRD patterns of photocatalysts obtained by using different alcohols (selected on the basis of the highest antibacterial activity tests).

During the studies photocatalysts with different amount of carbon and nitrogen were prepared. Presents of carbon should have influenced of absorption capacity of the materials and presents of nitrogen should have influenced on activity under visible light irradiation and also on the disinfection ability. Different crystallographic structure a different amount of carbon and nitrogen in the photocatalysts has been achieved by using alcohols with 1; 2 and 3 carbon groups (methanol, ethanol and isopropanol). The full characteristics of titania powders that were introduced into concrete powder are listed in Table 1. In Figure 1 and Figure 2, the XRD patterns of prepared photocatalysts are presented. 

As can be seen from Table 1, the temperature of modification strongly impacts on the crystalline phase of modified TiO_2_. It was possible to observe the anatase-to-rutile phase transformation possible at 600 °C, which was also confirmed in Figure 1 analyzing the XRD patterns of TiO_2_-N,C photocatalysts modified in the atmosphere of methyl alcohol (exemplary materials). The growth of anatase crystallites was also noted (from 10 to 28–34 nm after calcination at 600 °C). What is more, co-modification of TiO_2_ with nitrogen and carbon did not influence the phase composition, crystallite size or BET specific surface area (similar results were found for TiO_2_ samples modified with nitrogen). For all studied samples, the S_BET_ values decreased as the modification temperature increased, mainly due to the growth of crystallites size and the presence of rutile. Summarizing, it can be clearly stated that the modification temperature significantly affected the structural properties. 

It is possible to observe the differences in the amount of carbon and nitrogen. When the ethanol and isopropanol was used for modification whit the increasing of modification temperature the amount of nitrogen and amount of carbon also decreasing. There is different situation when methanol was used for modification, changing in modification temperature (100; 300; 600 °C) doesn’t have influence in amount of nitrogen in these catalysts and the amount of nitrogen is almost on the same level and amounted to (1.11; 1.05 and 1.18 N wt.% respectively)

**Figure 3:**
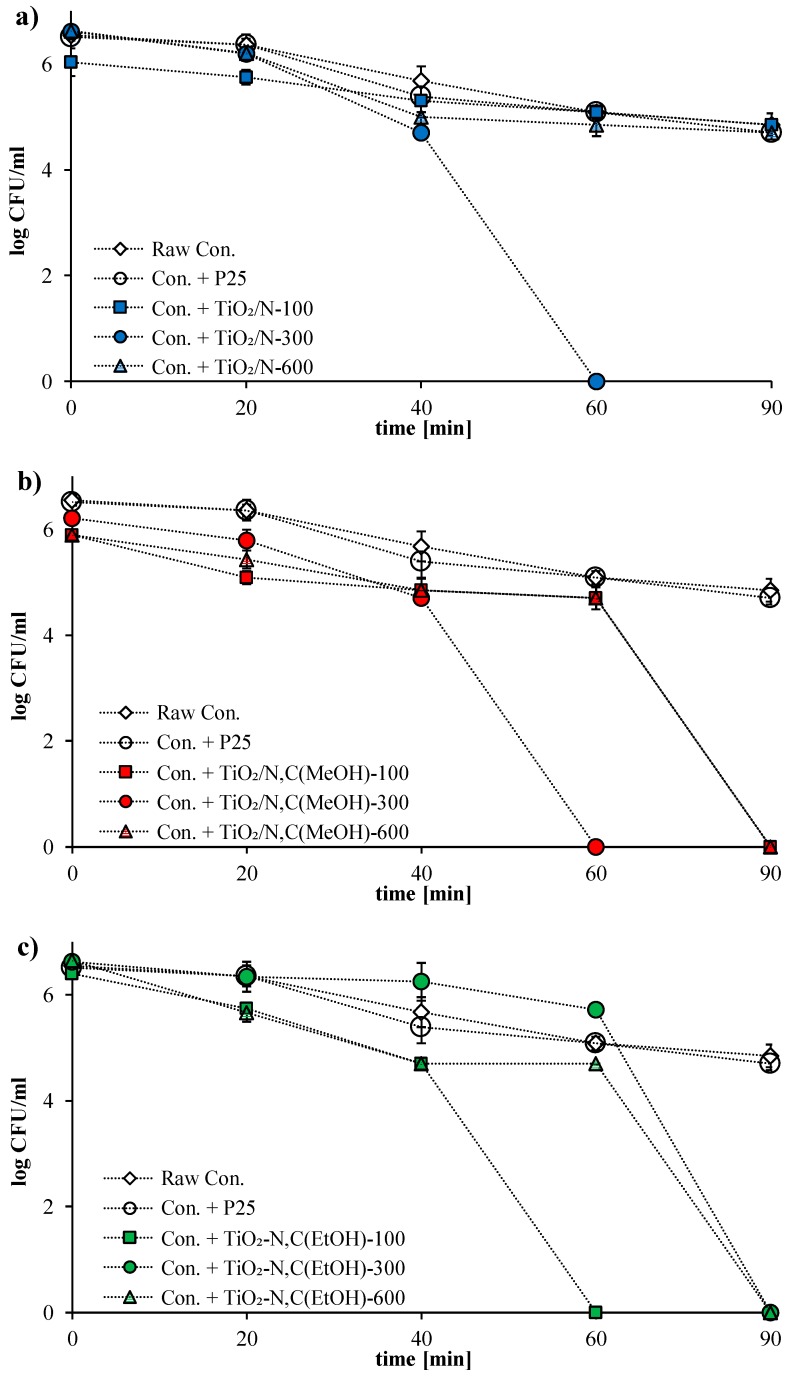

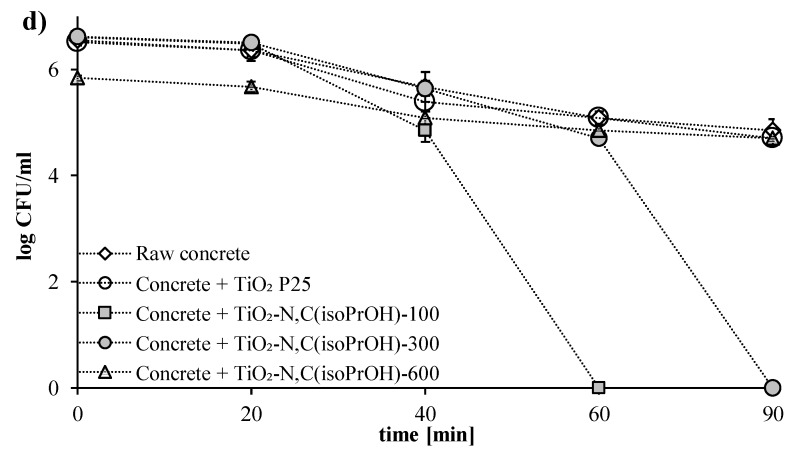
Inactivation rate of *E. coli* bacteria on the raw concrete plates, plates implemented commercial TiO_2_ P25 and (**a**) TiO_2_/N photocatalysts, (**b**) TiO_2_/N,C_MeOH_, (**c**) TiO_2_/N,C_EtOH_ and (**d**) TiO_2_/N,C_iso-PrOH_ under artificial solar light irradiation.

It is clearly demonstrated in Figure 3 that mixing of concrete cementitious powder with commercial titania TiO_2_ P25, N-TiO_2_ and co-modified N,C-TiO_2_ photocatalysts exhibited different antimicrobial potential. The detailed results of *E. coli* inactivation of concrete plates prepared from raw Concrete Fix M-15 Kreisel with and without (control experiment) the addition of titania powders, in dark conditions, as well as under artificial UV-Vis irradiation, are presented in Table 2 and Table 3, respectively. It was demonstrated that after 60 min of bacteria incubation in darkness or light conditions, about 20% of bacteria cells were removed. According to the procedure proposed by Guo [5], it is highly likely that bacteria cells were quickly washed away from the surface of the concrete plates. On the other hand, the porous structure of concrete plates contributes to the accumulation of bacteria cells within the pores [19]. This explains the reduced number of bacteria cells after 60 and 90 min of incubation. The proposed method should be considered for reference purposes only.

Figure 3 presents results for *E. coli* inactivation in the presence of concrete plates loaded with commercial TiO_2_ P25 and tested photocatalysts radiated under an artificial solar light source. 

Table 3 presents the removal rates of *E. coli* bacteria cells under artificial solar light. It was found that for concrete plates loaded with 10 wt.% of commercial TiO_2_ P25 around 56% of bacteria cells were killed after 90 min of UV-Vis irradiation. However, this does not mean that TiO_2_ P25 was photoexcited by visible light. The lamp used as a light source emits around 5% of UV-A light. It should be pointed out that TiO_2_ P25 was excited with UV light, which was also reported by Guo [20].

All concrete plates supplemented with modified by carbon and nitrogen TiO_2_ photocatalysts present much higher activity in comparison with TiO_2_ P25-implemented plates. It can be generally stated that nitrogen as well as nitrogen and carbon co-modification of TiO_2_ results in the enhancement of antimicrobial properties of tested building materials under UV-Vis light. Barbieriková et al [21] tested N-doped titanium dioxide nanosheets and found that upon UV photoexcitation the N-doped TiO_2_ nanopowders dispersed in water or in dimethlsulfoxide effectively generate reactive oxygen species (ROS) (hydroxyl radical, superoxide radical anion) detected as the corresponding 5,5-dimethyl-1-pyrroline N-oxide spin-adducts. The VIS-light-induced ROS formation was significantly lower, while the UV photoexcitation of N-doped TiO_2_ nanosheets dispersed in water led to the efficient generation of hydroxyl radicals. The visible-light photoactivation pathway involves the excitation generating species and photoelectrons as the key player in the ROS production. In the studies of Pan et al. [22] the ROS generated by N-TiO_2_-phthalocyanine, TiO_2_-phthalocyanine and phthalocyanine in aqueous suspensions under visible light irradiation were monitored by different ROS-sensitive fluorescence probes. The fluorescence intensities indicated the production of total ROS, •O_2_^−^/H_2_O_2_, and OH•, respectively.

It is very well known that carbon and/or nitrogen modification of TiO_2_ also leads to narrowing of titania band energy, thus, getting the TiO_2_ red shift to the visible light. In our earlier work UV-Vis/DR spectra of photocatalysts modified by ammonia and methanol at 100, 300 and 600 °C were presented. With the increasing of modification temperature pf photocatalysts the decreasing of absorption spectra in visible region can be observed. Moreover in the case of photocatalysts modified at 300 °C additional absorption spectrum in visible region is presented [23]. The highest activity in *E. coli* inactivation was found for N,C co-modified samples (see Figure 3b–d and Table 3), so it can be concluded that co-modification with N,C dopants is more effective than nitrogen modification. 

Another interesting aspect of the presented results was the role of calcination temperature in preparation of N-doped and N,C co-doped photocatalysts. Calcination of TiO_2_ causes morphological and structural changes. The physicochemical properties of titania-based powders are listed in Table 1. It was shown that higher temperature corresponds to the formation of larger crystallites. The effect of annealing temperature on the structural properties has been presented by many authors [24,25]. Some researchers stated that the crystal structure, crystal size, surface property and photocatalytic activity greatly influence by the annealing temperature. It has also been proven that a photocatalyst prepared without annealing will be mainly amorphous, whereas titanium dioxide crystal can be obtained by annealing treatment.

The antibacterial activity of a photocatalyst may also depend on the composition ratio of anatase to rutile. The difference could be due to variation in the photocatalytic activity between the above-mentioned phases. An enhanced antimicrobial effect of TiO_2_ in combination with UV-A light was observed for anatase form [26,27,28]. However, it is possible to attribute the antibacterial activity caused by another light source, such as sunlight or visible light to the photocatalyst modified by suitable dopants [10,27]. Our results have confirmed that TiO_2_ modified with nitrogen or nitrogen and carbon presented some antimicrobial properties under artificial solar irradiation. 

Also, the presents of nitrogen groups have influence of disinfection process. Zuo et al. [29] found that ${\mathrm{NH}}_{4}^{+}/{\mathrm{NH}}_{3}$ and ${\mathrm{NO}}_{2}^{-}$ inhibited obviously the photocatalytic disinfection process. Photocatalysts modified at 100 °C had the highest amount of nitrogen, and also in the case of photocatalysts modified by ethanol and isopropanol 100% bacterial removal rate was achieved by 60 min of irradiation.

The porous structure of concrete plates contributes to the accumulation of bacteria cells within the pores [19] and encourages the photocatalytic process. It has been widely accepted that the microorganism inactivation utilising TiO_2_ is based on the same photocatalytic oxidation mechanism like in case organic compound. The light activation of titania resulting in ROS production that may break down the bacterial cell wall and outer membrane peroxidation. This would allow cell contents to leak out and smaller TiO_2_ particles to enter, thereby promote photocatalytic reactions inside cells. The killing mechanism implies oxidation of the oxidative enzymes or other critical cell components and proteins or DNA. During this period the inactivation of bacteria could also be intoxicated due to the formation of by-products, such as acids or aldehydes. This process leads to precipitation of bacteria death [26]. 

The photocatalysis not only successfully inactivates bacteria cells but also decomposes the residual damaged cells. It was shown that during the first 20 min of irradiation, the bacterial inactivation was very slow (see Table 2). This is consistent with results obtained by Benabbou [30]. The main reason is the gradual oxidation of the bacterial membrane, which triggers the self-defence and auto-repair mechanism in bacterial cells. After 60 min, the anti-stress enzymes are no longer able to protect the bacterial membrane against oxidation. 

The disinfection kinetics of *E. coli* bacteria were also calculated. Four classical disinfection models were checked: Chick–Watson, modified Chick-Watson, Hom and modified Hom model. In Table 4 and Table 5, the R^2^ coefficient and kinetic constants calculated from the kinetic analyses of four models for *E. coli* photocatalytic oxidation under artificial solar light on concretes modified by addition of different types of photocatalysts are presented.

As it can be seen the best fit of data was to modified Hom model. 

**Figure 4:**
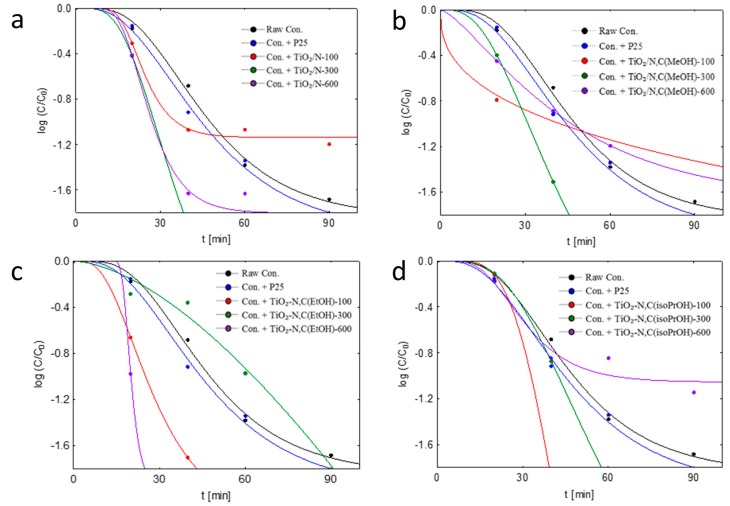
Fitting of the modified Hom kinetic model to experimental data of the photocatalytic inactivation of bacteria in suspension with tested raw concrete plates, plates implemented commercial TiO_2_ P25 and (**a**) TiO_2_/N photocatalysts, (**b**) TiO_2_/N,C_MeOH_, (**c**) TiO_2_/N,C_EtOH_ and (**d**) TiO_2_/N,C_iso-PrOH_ under artificial solar light radiation.

The result with the highest coefficient of determination was chosen as the best fitted and non-linear multivariate regression analysis with respect to the bacterial strains was used to determine the inactivation kinetics. On the basis of values of determination coefficients calculated for four models typical for *E. coli* inactivation, the modified Hom disinfection kinetic model was chosen and presented in Figure 4. As it can be seen, three different regions can be identified in the plot [31]: (i) an initial delay or smooth decay at the beginning of the reaction, usually called “shoulder”, (ii) log-linear inactivation region that covers most part of the reaction and (iii) a deceleration of the process at the end of the reaction, usually called “tail”. Marugán et al. [31] have used a similar modified Hom model for the photo-disinfection kinetic modelling of E. coli using standard P-25 TiO_2_. According to the photocatalytic disinfection mechanism proposed by Sunada et al. [32], the ROS generated upon irradiation of the semiconductor particles causes the bacterial inactivation by producing the partial decomposition of the external membrane, changing its permeability or destroying it, allowing the ROS to reach the cell wall and the cytoplasmic membrane, leading to the lysis of the cell. Consequently, bacteria are inactivated because of the cumulative effects of serial ROS attacks on the cell membrane-wall system.

The length of the shoulder region in the inactivation curves would depend on the volumetric rate of ROS generation, conditioned by the concentration of the semiconductor in the aqueous suspension and by the incident radiation flow, both factors determining the volumetric rate of photon absorption [31].

## 3. Experimental

### 3.1. Preparation of the N and/or C-Modified Titania Photocatalysts

In all experiments, commercial TiO_2_ (Grupa Azoty Zakłady Chemiczne “Police” S.A., Police Poland) was applied as a starting material in the preparation process of the TiO_2_-N and co-modified TiO_2_-N,C photocatalysts. The gaseous ammonia (Messer Polska Sp. z o.o., Szczecin, Poland) was used as a nitrogen source. The aliphatic alcohols methanol, ethanol and isopropanol (Avantor Performance Materials Poland S.A., Gliwice, Poland) were used as a carbon source. Gaseous argon with a gas grade purity of 99.9995% (Messer Polska Sp. z o.o., Poland) was used as an inert gas. All chemicals were used without further purification. 

The quartz crucible containing 15 g of starting TiO_2_ (predominantly anatase) was placed inside a quartz tube in a tubular furnace (Nabertherm GmbH, Lilienthal, Germany) and was heated up to the programmed temperature (100, 300 or 600 °C) in argon gas flow (100 mL/min, inlet pressure 2.5 bar). The titania sample was kept at an appropriate temperature for 1.5 h. For TiO_2_-N and TiO_2_-N,C photocatalysts, in the point of maximal temperature the argon flow was closed. The gaseous ammonia with a flow of 200 mL/min (inlet pressure 1.5 bar) was passed directly through quartz tube or Dreschel gas washing bottle containing appropriate kind of aliphatic alcohol, respectively. The photocatalysts were calcined for 1.5 h. The gas flow of both argon and ammonia was controlled by means of Mass-Stream mass flow controllers (M+W Instruments GmbH, Germany). The commercial AEROXIDE^®^ TiO_2_ P25 (Evonik Industries AG, Düsseldorf, Germany) was used as a reference photocatalyst.

### 3.2. Preparation of Concrete Plates

Concrete plates were obtained by mechanical grinding of concrete powder (Concrete Fix M-15 Kreisel) with 10 wt.% of the appropriate photocatalyst in agate mortar during 30 min [33]. The prepared mixture was blended with water (water-to-concrete ratio *w/c* = 0.43). Obtained homogenous pastes were poured into silicone moulds (20 × 20 × 6 mm) and dried at room temperature (25 °C) until a constant mass was formed. After hardening, the samples were taken out of moulds and dried for 28 days at room temperature.

### 3.3. Photocatalysts Characterisation

The nitrogen adsorption/desorption isotherms for determining the values of the specific surface areas (S_BET_) for obtained photocatalysts were determined using Quadrasorb SI analyser (Anton Paar GmbH, Graz, Austria, previously Quantachrome Instruments, Boynton Beach, FL, USA). The crystallite size and crystal structure of obtained photocatalysts were analysed by the X-ray diffraction analyses (XRD) carried out with PANalytical Empyrean X-ray diffractometer (Enigma Business Park Grovewood Road United Kingdom) equipped with Cu Kα radiation (λ = 0.154056 nm). The total carbon and nitrogen content in TiO_2_-N as well as co-modified TiO_2_-N,C photocatalysts was determined by a Multi N/C carbon analyser equipped with a high-temperature combustion system HT 1300 solid module (Analytic Jena, Jena, Germany) and ONH 836 elemental analyser (Leco Corporation, St. Joseph, MN, USA).

### 3.4. Determination of the Antimicrobial Properties of Concrete Plates

**Figure 5:**
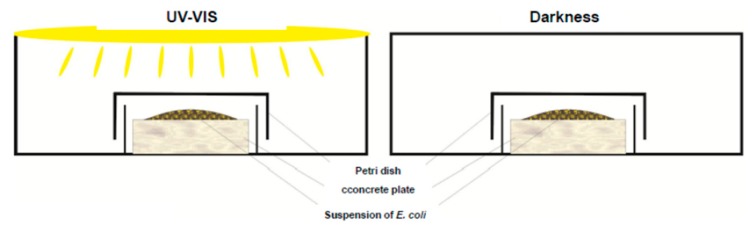
Schematic experimental setup.

The antimicrobial activity of materials was evaluated against *Escherichia coli* K12 (ATCC 25922). *E. coli* was precultured and maintained on Enriched Broth (BioMaxima S.A., Lublin, Poland) at 37 °C for 24 h. To eliminate the liquid medium, the bacteria cells were separated by centrifugation at 4000 rpm for 10 min. After that, the bacterial cell pellet was resuspended and diluted in sterilised 0.9% (*w/v*) sodium chloride solution to the concentration 0.5 in McFarland standard (BioMérieux, Craponne, France), approximately 1.5 × 10^8^ CFU/mL. 1 mL of *E. coli* cell suspension was pipetted onto each concrete sample, which was placed in a sterilised Petri’s dish (9.0 cm diameter) made of quartz glass to prevent drying and to provide visible light transmission (Figure 5). The experiments were carried out under artificial solar light (one bulb 300 W, OSRAM Ultra Vitalux, Warszawa, Poland) placed about 15 cm from the concrete sample. 

**Figure 6:**
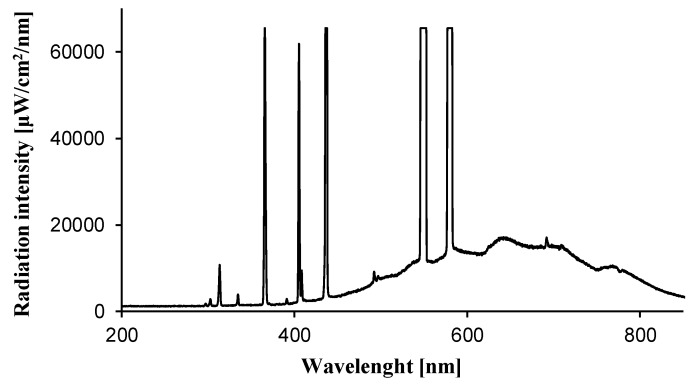
Emission spectra of the artificial solar light source (Ocean Optics Inc., Largo, FL, USA).

The emission spectra presented in Figure 6 was measured by using Ocean Optics USB 4000 spectrometer (Ocean Optics Inc., Largo, FL, USA). The radiant flux was monitored with a radiation intensity meter LB901 (Lab-EL, Reguły, Poland) equipped with CM3 and PD204AB Cos sensors. The radiation intensity of the utilised light source was 9.0 W/m^2^ in the spectral range from 300 to 2800 nm and 258.1 W/m^2^ in the spectral range from 280 to 380 nm. After irradiation, the cell suspension was collected by washing the sample with 20 mL of 0.9% sodium chloride solution at different time intervals of 20, 40, 60, 90 and 120 min, respectively. Then, serial decimal dilutions of the collected suspension were performed. 0.25 mL of each diluted suspension was spread over the surface of Plate Count Agar PCA (BioMaxima S.A., Lublin, Poland) and incubated at 37 °C for 18 h. The viable colony were counted and showed as the colony-forming units (CFU/mL). In all experiments, control tests in dark conditions were carried out simultaneously. Disinfection efficiency on concrete plates without TiO_2_ irradiated under artificial UV-VIS was also performed. All the equipment and media were autoclaved at 126 °C for 13 min before the experiment to ensure sterility. All experiments were carried out at ambient temperature (25 ± 3 °C).

### 3.5. Kinetics of the Photocatalytic Disinfection

Additionally, the disinfection kinetics of *E. coli* in the presence of tested concrete plate samples were calculated. Four classical disinfection models were checked: Chick–Watson (Equation (1)), modified Chick–Watson (Equation (2)), Hom (Equation (3)) and modified Hom model (Equation (4)). The equations are expressed as follows [31]:

(1) $$\frac{\log C}{\log C_{0}}=-kt$$

where *C* is the bacteria concentration [CFU/mL] at time *t* [min], *C*_0_ -is the initial bacteria concentration [CFU/mL], and *k* represents the photocatalytic destruction kinetic rate constant;

(2) $$\frac{\log C}{\log C_{0}}=-k_{1}\left[1-\exp\left(-k_{2}t\right)\right]$$

where the kinetic rate constant *k* has divided into two parameters expression, that is, *k*_1_ and *k*_2_;

Hom [34] proposed the empirical Hom model after it was observed that the survivor plots of natural algal–bacterial systems were curvilinear, rather than typical log-linear type

(3) $$\frac{\log C}{\log C_{0}}=-kN^{n}t^{h}$$

where: $\frac{\log C}{\log C_{0}}$ bacterial reduction unit; *C* is the bacterial population at time t; *C*_0_ is the initial bacterial population; k = experimental reaction rate, *N* = concentration of photocatalyst used, t is the applied irradiation time, *n, h*—empirical parameters. This two-parameter empirical model predicts the bacterial inactivation in a non-linear function for both *N* and *t* and is directly dependent on the model parameters *n* and *h*, respectively. Owing to the two-parameter nature of the model, it can only account for either shoulder or tailing characteristics but not both. A modification (Equation (3)) to incorporate an additional empirical parameter was made to accurately account for the three different inactivation characteristics

(3) $$\frac{\log C}{\log C_{0}}=-k_{1}{\left[1-\exp\left(-k_{2}t\right)\right]}^{k_{3}}$$

where: *k*_1_, *k*_2_ and *k*_3_ is the empirical constants for the modified Hom model.

Analysis of obtained results was conducted using Statistica 13.3 software (StatSoftPolska, Kraków, Poland).

## 4. Conclusions

The results showed that the presence of TiO_2_ particles increased the antibacterial properties of modified concretes. It was concluded that the bacteria destruction process begins from the cell wall and proceeds towards intracellular components, which is typical for the photocatalytic inactivation of microorganism’s cells. The type of photocatalyst introduced into the concrete plates plays a decisive role in determining the structure of the final product and is a key factor influencing the bacterial removal rate. Nitrogen and carbon co-modification of starting TiO_2_ mainly enhanced the antibacterial activity and prepared photocatalysts were capable of total *Escherichia coli* K12 inactivation under artificial solar light. The modified Hom model of disinfection kinetic models described the best-fit model for the obtained results. The concretes supplemented with modified titania can be widely used in places with demanding high sterilisation levels such as hospitals, institutions, schools, etc., or for construction water storage tanks.

## Figures and Tables

**Table 1:** Physicochemical characteristic of reference TiO_2_ P25 and modified TiO_2_ photocatalysts.

Sample Code	S_BET_ [m^2^/g]	Crystallite Size * [nm]	Crystallite Contribution [%]		Nitrogen Content [wt.%]	Carbon Content [wt.%]
		Anatase	Anatase	Rutile		
TiO_2_ P25	55	21	80	20	-	-
TiO_2_/N-100	259	10	97	3	0.57	-
TiO_2_/N-300	158	13	95	5	0.31	-
TiO_2_/N-600	34	28	87	13	0.05	-
TiO_2_/N,C_MeOH_-100	277	10	98	2	1.11	1.6
TiO_2_/N,C_MeOH_-300	161	12	98	2	1.05	0.7
TiO_2_/N,C_MeOH_-600	29	34	83	17	1.18	0.06
TiO_2_/N,C_EtOH_-100	269	10	97	3	0.93	1.02
TiO_2_/N,C_EtOH_-300	168	13	97	3	0.77	0.87
TiO_2_/N,C_EtOH_-600	23	33	88	12	0.39	0.04
TiO_2_/N,C_isoPrOH_-100	265	10	98	2	1.17	0.97
TiO_2_/N,C_isoPrOH_-300	166	13	97	3	0.32	0.26
TiO_2_/N,C_isoPrOH_-600	30	33	85	15	0.12	0.05

* average crystallite size of anatase according to Scherrer’s equation.

**Table 2:** The reduction [%] of initial bacteria cells number in the darkness.

Samples				Time [min]			
				20	40	60	90
Samples Code			Calcination Temperature [°C]	Bacteria Removal Rate [%]			
Raw concrete			-	16.1	13.8	19.8	31.6
Concrete + TiO_2_ P25			-	8.5	9.5	13.8	17.7
Concrete + TiO_2_	N	-	100	2.7	15.4	24.9	32.3
		-	300	10.3	18.6	47.5	57.1
		-	600	0.3	39.9	43.1	45.3
		C_MeOH_	100	9.6	34.0	36.9	51.9
			300	13.4	40.3	47.3	52.1
			600	2.1	10.5	31.1	44.5
		C_EtOH_	100	12.6	39.2	42.6	52.2
			300	11.4	45.4	100	100
			600	4.3	7.7	29.4	100
		C_isoPrOH_	100	6.3	33.5	34.7	40.5
			300	11.7	32.8	38.0	41.0
			600	1.4	12.5	34.7	43.1

**Table 3:** The reduction [%] of initial bacteria cells number under artificial solar light.

Samples				Time [min]			
				20	40	60	90
Samples Code			Calcination Temperature [°C]	Bacteria Removal Rate [%]			
Raw concrete			-	1.6	12.6	18.6	41.7
Concrete + TiO_2_ P25			-	1.9	7.4	22.7	56.3
Concrete + TiO_2_	N	-	100	8.8	32.8	44.9	53.2
		-	300	6.3	42.2	100	100
		-	600	2.3	38.1	46.4	61.0
		C_MeOH_	100	11.8	21.1	46.2	100
			300	3.9	50.8	100	100
			600	5.4	36.2	52.8	100
		C_EtOH_	100	11.4	45.4	100	100
			300	6.3	18.3	35.9	100
			600	21.0	53.5	53.3	100
		C_isoPrOH_	100	9.8	34.8	100	100
			300	3.6	24.4	49.9	100
			600	2.9	32.1	34.5	56.9

**Table 4:** R^2^ value obtained from the kinetic analyses of tested models.

	R^2^ Value			
Sample Code	Chick-Watson	Modified Chick-Watson	Hom	Modified Hom
Raw concrete	0.951	0.950	0.995	0.994
Concrete + TiO_2_ P25	0.963	0.962	0.964	0.995
Concrete + TiO_2_/N-100	0.777	0.918	0.884	0.993
Concrete + TiO_2_/N-300	0.885	0.880	0.996	0.999
Concrete + TiO_2_/N-600	0.821	0.917	0.821	0.985
Concrete + TiO_2_/N,C(MeOH)-100	0.817	0.962	0.842	0.984
Concrete + TiO_2_/N,C(MeOH)-300	0.916	0.915	0.997	0.999
Concrete + TiO_2_/N,C(MeOH)-600	0.990	0.998	0.997	0.999
Concrete + TiO_2_-N,C(EtOH)-100	0.980	0.978	0.998	0.999
Concrete + TiO_2_-N,C(EtOH)-300	0.884	0.883	0.934	0.998
Concrete + TiO_2_-N,C(EtOH)-600	0.870	0.968	0.952	0.997
Concrete + TiO_2_-N,C(isoPrOH)-100	0.757	0.766	0.998	0.999
Concrete + TiO_2_-N,C(isoPrOH)-300	0.864	0.863	0.995	0.999
Concrete + TiO_2_-N,C(isoPrOH)-600	0.889	0.921	0.909	0.961

**Table 5:** Kinetic constants obtained from the kinetic analyses of four models for *E. coli* inactivation under artificial solar light radiation.

		Kinetic Constants							
	Kinetic Model	Chick-Watson	Modified Chick-Watson		Hom		Modified Hom		
Sample Code		K	k_1_	k_2_	k	h	k_1_	k_2_	k_3_
Raw concrete		0.0193	17.032	0.0011	0.0121	1.1041	1.8401	0.0471	5.6751
Concrete + TiO_2_ P25		0.2073	25.048	0.0008	0.0156	1.0051	1.9732	0.0422	4.0911
Concrete + TiO_2_/N-100		0.0161	1.426	0.0231	0.0957	0.6789	1.1380	0.1457	23.271
Concrete + TiO_2_/N-300		0.0425	178.97	0.0002	0.0006	2.1893	3.2827	0.0608	5.8513
Concrete + TiO_2_/N-600		0.0252	2.442	0.0188	0.1112	0.6471	1.8033	0.1238	16.677
Concrete + TiO_2_/N,C(MeOH)-100		0.0219	1.191	0.0472	0.2377	0.3834	2.8938	0.0016	0.3945
Concrete + TiO_2_/N,C(MeOH)-300		0.0342	157.65	0.0002	0.0012	1.9572	3.267-	0.0418	3.7096
Concrete + TiO_2_/N,C(MeOH)-600		0.0297	2.936	0.0086	0.0369	0.8511	1.6732	0.0268	1.4654
Concrete + TiO_2_-N,C(EtOH)-100		0.0406	107.10	0.0003	0.0113	1.3636	2.2023	0.0720	4.4654
Concrete + TiO_2_-N,C(EtOH)-300		0.0139	55.863	0.0002	0.0009	1.6813	7.9963	0.0012	1.6725
Concrete + TiO_2_-N,C(EtOH)-600		0.0383	2.449	0.0396	0.2102	0.5617	1.9543	0.4301	33.760
Concrete + TiO_2_-N,C(isoPrOH)-100		0.0392	212.19	0.0018	0.0001	3.9712	9.7661	0.0408	7.5177
Concrete + TiO_2_-N,C(isoPrOH)-300		0.0271	129.76	0.0002	0.0003	2.1056	3.6687	0.0360	5.3031
Concrete + TiO_2_-N,C(isoPrOH)-600		0.0139	1.869	0.0107	0.0370	0.7920	1.0558	0.0812	7.7489
